# Behaviors and Culture of Drinking among Korean People

**Published:** 2018-07

**Authors:** Seungduk KO, Aeree SOHN

**Affiliations:** 1. Dept. of Health Management, Hyupsung University, Suwon City, South Korea; 2. Dept. of Health Management, Sahmyook University, Seoul, Korea

**Keywords:** Drinking, Culture, Drinking behavior, Koreans

## Abstract

**Background:**

The objective of this study was to identify behaviors and culture of drinking alcohol in Korean people.

**Methods:**

Among a panel of subjected enrolled in existing domestic survey companies, adults aged 19 to 59 yr old who replied that their drinking frequency was more than once a month for the past one year were selected in 2017. Sample size and methods used for analysis were determined by considering demographically proportioned stratified sampling and monthly alcohol drinking rate. A total of 1,185 subjects (731 males and 454 females) responded to questionnaires.

**Results:**

Most drinking behaviors were in the domain of leisure time. Drinking for two or more times a month and binge drinking were mainly concentrated in five occasions types: going out with friends (44.2%), going out with colleague (34.2%), drinking at home or friend’s home with friends (32.9%), drinking at home alone (29.3%), and drinking with meals at home (27.5%). Regarding the ratio of experiences in drinking for subjects according to occupations, ‘Directors/Managers’ who participated in receptions of guests, either hosted by or invited thereto, had the highest percentage (64.2%), followed by ‘Individual Proprietors’ (56.4%). Differences between each type of occupation were found to be statistically significant (*P*< 0.001).

**Conclusion:**

The drinking culture in Korea was characterized by more social drinking than by drinking alone at home. The drinking behavior was often one-shot at a time rather than drinking a little sip. The practices of collective drinking should be improved to avoid secondary harmful effects.

## Introduction

Alcoholic drinks, together with tobacco, have been regarded as choices of one’s preference. Thus, drinking alcoholic beverage or selecting preferred drinks depends on one’s taste and one’s individual life style. However, in terms of behaviors involving drinking such as when, how, and what kinds of spirits do people drink, drinking is a social behavior determined by the society that people live in (1). The notion of alcohol and behaviors of drinking recognized by groups in a society acts as a sort of social norm. It influences drinking behaviors (2, 3). Alcoholic drinks are materials endowed with social significance (1). They are presented in religious rituals, rites of passage, and ceremonial occasions beyond its original function of intoxication. People drink alcoholic liquors when they feel happiness or sadness. They also drink when they are in ceremonial occasions such as the coming-of-age ceremony, marriage, ancestor memorials, and so on. Thus, behaviors of drinking alcoholic contents reflect history, culture, religion, living habit, and racial characteristics of each ethnic group. The drinking culture of an individual country is developed in a way to reflect unique social norms and life styles in each society. The attitude or behavior of drinking of an individual constitutes one’s social behavior involving others irrespective of the style of drinking (alone or together with others). It is a social fact generated in social relations wherein individuals interact with each other (1, 4).

Motives behind drinking of alcohol in different countries may be similar. However, attitudes toward drinking or behavioral styles (manners) of drinking alcohol are significantly different depending on religions and cultures of each country (5). The way of drinking alcohol signifies characters of each society. Because of this, alcoholic drinks are referred to as ‘foods of society’ (1). Some religions such as Islam, Buddhism, Mormonism, and Seventh-day Adventist Church prohibit drinking of alcohol by respective religious doctrines. If such religions are taken as national ones, then such countries will not sell alcoholic drinks. Societies thereof will not have groggeries/pubs or bars either. Thus, topics involving the culture of drinking alcohol should be discussed with perspectives of religions, value systems, social norms, social structures, and social relations to understand drinking behaviors of people. Expectancies and attitudes around drinking are important individual-level factors that influence choices about alcohol, patterns, and outcomes (6). Alcohol expectancies are the beliefs individuals hold about the likely positive or negative outcomes that will result from their drinking, and are closely linked with behavior (7). Habitual practices such as how and when people would take alcoholic drinks have changed constantly in different ages and times. In the past, there were occasions of drinking hosted by litterateurs. In contemporary age, there are receptive cultures accompanying the drinking of alcoholic drinks hosted by business people. Thus, practices such as drinking alcoholic beverage with refreshments in the fields, taking alcoholic drinks to titillate the fun in ceremonies of each community, and drinking in the memorials of ancestors frequently found in agricultural societies are currently stagnated. In this way, aspects of lives in each society are reflected in behaviors of people drinking alcohol. Recently, the concern about diverse types of harmful effects resulting from excessive drinking of alcoholic beverage has been growing continuously in our society. This necessitates correct analyses on how and why people would drink alcoholic drinks to identify the nature of such concern. Such analyses will lead us to substantive realities of our society.

A number of studies have been carried out to identify factors of alcoholism and/or identification/prevention of problems due to heavy drinking (8–11). However, the Korean studies on issues involving the identification of how domestic people would drink under what culture thereof, their thoughts on alcoholic drinks, and their behaviors with social norms of drinking are relatively few. Most previous studies have focused on limited issues of alcoholism or problems thereof (10, 11). In Europe countries including the United Kingdom, various active studies focusing on issues related to drinking behavior and drinking social norms are frequently conducted. Much attention has been paid to the relative impact of various factors that influence drinking behavior. Issues related to drinking behaviors or drinking norms in domestic society have already been addressed in press releases or articles in mass media. However, data from national probability samplings are quite limited and deflected.

Thus, the objective of this study was to identify substantive realities of drinking alcohol in aspects of ‘the significance of alcoholic drinks’ perceived by domestic people and ‘how alcoholic drinks are taken’ by domestic people selected through probability sampling.

## Methods

### Data Collection

To determine the number of samples of subjects as participants of this study, demographically proportioned stratified sampling was carried out using a panel of subjects enrolled in a domestic survey company M in Nov 2017. From this panel, target number of subjects according to sex and age was determined by taking monthly drinking rate of people reflected in the “6^th^ Korea National Health and Nutrition Examination Survey (12)” (Table 1) into account. Those with age in their 20s to 50s with drinking frequency of more than once a month were selected as subjects of this study.

**Table 1:** Numbers of Planned vs. Actual Samples determined by considering sex, age, and drinking frequencies of subjects

	***Male Subjects***				***Female Subjects***			
***Age (yr)***	***Ratio of Population***	***Monthly drinking rate***	***Planned Number of Samples***	***Actual Number of Samples***	***Ratio of Population***	***Monthly drinking rate***	***Planned Number of Samples***	***Actual Number of Samples***
20s	0.23	0.769	164	162	0.21	0.566	106	111
30s	0.24	0.800	183	186	0.24	0.506	107	107
40s	0.27	0.793	202	204	0.28	0.529	130	131
50s	0.26	0.744	183	179	0.27	0.421	102	105
Total			732	731			445	454

A total of 1185 subjects were sampled for this study, including 731 male subjects and 454 female subjects. Before conducting the survey with the prepared questionnaire, the protocol of this study was approved by our Institutional Review Board (2-104078-AB-N-01-2017105HR). The survey was carried out from Nov 1st, 2017 to Nov 8th, 2017 through on-line communication. A total of 1843 individuals were contacted with the questionnaire through on-line communication and 1222 subjects retuned responses to the questionnaire (575 were unqualified respondents, 37 subjects dropped out from midway). After excluding 37 subjects with incomplete responses, the remaining 1185 subjects with complete responses were then selected as participants in this study.

### Measurements

#### Occupation of Subjects

Occupations of subjects had the following: ‘Professionals/Sedentary Workers’, ‘Directors/Managers’, ‘Sales (Service) Persons/Daily Laborers’, ‘Employees of Production/Transportation’, ‘Individual Proprietors’, and ‘Students/Unemployed/Retirees’. Occupations of these subjects were then classified into the following categories: ‘Professionals/Sedentary Workers’, ‘Directors/Managers’, ‘Salesperson/Daily Laborers’, ‘Workers in Production/Transportation Industries’, ‘Individual Proprietors’, and ‘Students/Unemployed/Others (Retirees)’.

#### Number of Drinking rounds and Drinking Culture in Receptions of Guests

Instead of meeting up in one place and staying there the entire night, groups of Korean friends will jump around from place to place in what is called “rounds” (cha in Korean). Sometimes it’s to change up the activity (i.e. restaurant for eating -> cafe for coffee) or simply just a change of venue (i.e. drinking at a bar -> drinking at a chicken joint). Whatever the case, be ready to move around. Thus, drinking rounds were distinguished into three cases of venues: drinking with meals, post-meal drinking, and drinking late at night or continued until dawn.

Drinking at receptions of guests signified that subjects were either invited to the reception or they hosted the reception.

#### Drinking Behaviors in Different Situations

To identify drinking behaviors of subjects, types of alcoholic drinks (all kinds of alcoholic drinks, including beer, wine, and soju (distilled spirits)) taken by each subject during the past one year were examined. Since types and amounts of alcoholic drinks are dependent on when and who participated in drinking occasions, the frequency of drinking and the amount of alcoholic drinks were examined at different situations: 1) Drinking at Ceremonial Occasions (Marriage, Funeral, Memorials), 2) Drinking with Family Meals, 3) Drinking at Home or at Friends’ Home, 4) Drinking when Dining with Friends or Acquaintances, etc., 5) Drinking when Dining with Colleagues, 6) Drinking in Groggeries/Pubs or Bars, and 7) Drinking Alone.

#### Binge Drinking

Regarding the definition of binge drinking (crapulence), subjects corresponded to the following conditions: drinking frequency of more than once a month and more than 7 standard drinks (male) or 5 standard drinks (female) at each drinking, irrespective of types of alcoholic drinks.

## Results

### Times for Drinking and Number of Drinking Rounds

From the inquiry delved into time of drinking, the time starting from 6 o’clock in the evening after completion of daily work of subjects was found to be the most frequent time employed for drinking. Regarding the frequency of time used for drinking after 6 o’clock according to weekdays and weekends, results were as follows: Friday evening, 74.6%; Saturday evening, 71.8%; Wednesday evening, 31.4%; Thursday evening, 31.1%; Sunday evening, 31.1%; Tuesday evening, 24.0%; and Monday evening, 20.4%. Daytime drinking was mostly found on Saturday (13.5% among entire subjects) and Sunday (9.9% among entire subjects). It occurred less on weekdays (1% ∼ 5% among entire subjects). Subjects in their 20s were found to mostly participate in the drinking after 6 o’clock on Saturday evenings whereas subjects belonging to other age groups mostly participated in the drinking on Friday afternoons (Table 2).

**Table 2:** Time zones and number of sessions for drinking at each age group

***Variable***	***20s (N=273) %***	***30s (N=293) %***	***40s (N=335) %***	***50s (N=284) %***	***Total (N=1195) %***
Time Zones for Drinking†					
Fri. After 6 o’clock P.M.	79.1	77.1	71.6	71.1	74.6
Sat. After 6 o’clock P.M.	84.2	75.4	68.1	60.6	71.8
Wed. After 6 o’clock P.M.	22.0	36.9	32.2	33.8	31.4
Thu. After 6 o’clock P.M.	28.9	37.2	26.9	32.0	31.1
Sun. After 6 o’clock P.M.	39.6	29.4	26.6	30.3	31.1
Tue. After 6 o’clock P.M.	17.2	27.3	24.5	26.4	24.0
Mon. After 6 o’clock P.M.	15.4	23.9	17.9	24.6	20.4
Sat. Before 6 o’clock P.M.	12.8	13.0	13.7	14.4	13.5
Sat. Before 6 o’clock P.M.	11.0	10.6	9.0	9.2	9.9
Fri. Before 6 o’clock P.M.	7.0	2.7	4.8	6.0	5.1
Wed. Before 6 o’clock P.M.	1.5	2.4	3.3	1.8	2.3
Thu. Before 6 o’clock P.M.	2.2	2.0	1.2	2.1	1.9
Tue. Before 6 o’clock P.M.	1.8	1.7	0.9	2.1	1.6
Mon. Before 6 o’clock P.M.	1.8	1.7	1.8	0.4	1.4
Rounds for Drinking***					
1st Round (Drinking with meals)	16.5	21.2	35.8	45.1	30.0
2nd Round (Post-meal Drinking)	53.1	63.5	56.1	48.9	55.5
3rd Round (Drinking late at Night or even until Dawn)	30.4	15.4	8.1	6.0	14.5

† Multiple Replies,

*** *P*<.001 (Mantel-Hanszel x^2^ test was carried out after controlling sex)

From subjects who replied to the inquiry on the number of drinking rounds they usually took, results for frequencies of drinking rounds were as follows: taking sole dinking session (30%), taking two drinking rounds (55.5%), and taking more than two drinking rounds (14.5%). Subjects in their 20s were dominant in the three rounds of drinking. Differences in numbers of drinking rounds between each age group were found to be statistically significant (Table 2).

### Situations coupled with Drinking Frequency over two per Month, Binge Drinking, and Average Number of Drinks

Table 3 shows situations coupled with drinking frequency of more than two per month, binge drinking rate, and average number of standard drinks. The situation coupled with the highest drinking frequency of more than twice a month was ‘Drinking while Dining with Friends or Acquaintances, etc.’, followed by ‘Drinking while Dining with Colleagues’ (34.2%), ‘Drinking at Home or at Friends’ Home’ (32.9%), ‘Drinking Alone’ (29.3%), ‘Drinking with Family Meals’ (27.5%), ‘Drinking in Groggeries/Pubs or Bars’ (20.8%), and ‘Drinking at Ceremonial Occasions (Marriage, Funeral, Memorials)’ (5.3%).

The subjects in the age group of 50s showed significantly higher portion of the situation of ‘Drinking with Family Meals’ compared to those in other age groups (*P*<.001). For subjects in the age group of the 20s, 54.6% of them showed ‘Drinking while Dining with Friends or Acquaintances etc.’ and 28.6% of them showed ‘Drinking in Groggeries/Pubs or Bars’. These portions were significantly higher than those in other age groups (*P*<.001). For subjects in the age group of the 30s, 35.2% had ‘Drinking while Dining with Colleagues’ and 30.4% of them showed ‘Drinking Alone’. These proportions were higher than those in other age groups. However, such differences between age groups were not statistically significant.

**Table 3:** Situations for drinking frequency of more than twice per month and average number of drinks

***Drinking Situations***	***Drinking Frequencies over 2 per Month (%)***					***Number of standard Drinks (Mean)***				
	**20s**	**30s**	**40s**	**50s**	**Total**	**20s**	**30s**	**40s**	**50s**	**Total**
Drinking at Ceremonial Occasions (Marriage, Funeral, Memorials)	5.5	4.1	5.1	6.7	5.3	2.0	2.1	2.3	2.6	2.3
	X^2^=1.98 (*P*=.576)					F=3.67 *P*=.012				
Drinking with Family Meals	17.6	28.7	29.9	33.1	27.5	2.5	3.0	2.7	2.7	2.7
	X^2^=20.17 (*P*=.000)					F=2.07 *P*=.102				
Drinking at Home or at Friends’ Home	33.7	33.4	31.3	33.5	32.9	4.2	4.3	3.8	3.3	3.9
	X^2^=0.53 (*P*=.913)					F=5.45, *P*=.001				
Drinking when Dining with Friends or Acquaintances etc.	54.6	43.0	39.4	41.2	44.2	5.6	5.5	4.9	4.8	5.2
	X^2^=16.19 (*P*<.001)					F=4.37, *P*=.005				
Drinking when Dining with Colleagues	34.8	35.2	32.2	34.9	34.2	4.8	5.4	4.9	4.8	5.0
	X^2^=0.79 (*P*<.851)					F=1.75, *P*=.154				
Drinking in Groggeries/Pubs or Bars	28.6	18.1	19.7	17.3	20.8	3.7	3.1	3.4	3.1	3.3
	X^2^=13.11 (*P*<.004)					F=1.78, *P*=.148				
Drinking Alone	30.0	30.4	29.6	27.1	29.3	2.9	2.9	2.7	2.5	2.7
	X^2^=.91 (*P*<.823)					F=1.20, *P*=.308				

The amount of alcoholic drinks consumed at each situation was examined. Results revealed an average of 5.5 standard drinks for ‘Drinking while Dining with Friends or Acquaintances etc.’ and 5.0 standard drinks for ‘Drinking while Dining with Colleagues’.

Overall, the situation of ‘Drinking while Dining with Friends or Acquaintances etc.’ was dominant over other situations. It also had higher mean number of standard drinks.

Subjects in the age group of the 50s were found to have a frequency of 33.1% for ‘Drinking with Family Meals’. This was significantly higher than the frequency of subjects in other age groups (*P*<.001). Besides, subjects in their 50s had a mean of 2.6 standard drinks for situation of ‘Drinking at Ceremonial Occasions (Marriage, Funeral, Memorials)’. This number for such situation was also significantly higher than subjects in other age groups (*P*<.001).

Table 4 shows occasions corresponding to the drinking frequency of more than once a month wherein more than 7 standard drinks for males or more than 5 standard drinks for females are taken (binge drinking), irrespective of types of alcoholic drinks. Results of frequency of binge drinking for each occasion of drinking are as follows: 74.9% for ‘Drinking when Dining with Friends or Acquaintances etc.’, 70.0% for ‘Drinking when Dining with Colleagues’, 61.2% for ‘Drinking at Home or at Friends’ Home’, 50.4% for ‘Drinking with Family Meals’, 45.2% for ‘Drinking Alone’, 45.0% for ‘Drinking in Groggeries/Pubs or Bars’, and 32.1% for ‘Drinking at Ceremonial Occasions (Marriage, Funeral, Memorials)’.

**Table 4:** Situations coupled with Binge drinking

***Drinking Situations***	***Binge Drinking (%)***				
	**20s**	**30s**	**40s**	**50s**	**Total**
Drinking at Ceremonial Occasions (Marriage, Funeral, Memorials)	28.2	30.4	31.3	38.4	32.1
	X^2^=7.42 (*P*=.060)				
Drinking with Family Meals	48.7	52.2	49.9	50.7	50.4
	X^2^=0.75 (*P*=.862)				
Drinking at Home or at Friends’ Home	63.4	62.8	60.3	58.5	61.2
	X^2^=1.82 (*P*=.599)				
Drinking when Dining with Friends or Acquaintances etc.*	79.9	76.8	69.6	74.6	74.9
	X^2^=9.20 (*P*=.027)				
Drinking when Dining with Colleagues	67.0	73.7	66.9	72.5	70.0
	X^2^=5.53 (*P*=.137)				
Drinking in Groggeries/Pubs or Bars**	52.4	36.2	45.7	46.1	45.0
	X^2^=15.56 (*P*=.001)				
Drinking Alone	49.5	46.1	44.5	41.2	45.2
	X^2^=3.99 (*P*=.262)				

Subjects in the age group of the 20s had binge drinking frequencies of 79.9% (*P*<.05) for ‘Drinking when Dining with Friends or Acquaintances etc.’ and 52.4% (*P*<.01) for ‘Drinking in Groggeries/Pubs or Bars’. These frequencies were significantly higher than those of subjects in other age groups.

### Experiences of Drinking at Receptions of Guests of Subjects in Respective Occupations and Undesirable Behaviors of Drinking

Table 5 shows results of inquiries delved into experiences of drinking at receptions of guests of subjects with respective occupations and undesirable behaviors of drinking. Regarding the portion of replies coupled with experiences of drinking at receptions of guests, the highest portion was 64.2% for subjects with occupations of ‘Directors/Managers’, followed by 56.4% for subjects in occupational category of ‘Individual Proprietors’. Differences in experiences between each occupational category were found to be statistically significant (*P*<0.001).

**Table 5:** Experiences of drinking at receptions and undesirable drinking behaviors of respective occupations

Drinking Receptions***		***Professionals***	***Directors/Managers***	***Salesperson/Daily Laborers***	***Workers in Production***	***Individual Proprietors***	***Students/Un employed/Others***	***Total***
	Yes	42.4	64.2	30.6	41.9	56.4	20.4	38.8
	No	57.6	35.8	69.4	58.1	43.6	79.6	61.2
Experiences of the Culture of Collective Drinking								
	Passive and Reluctant Participation in Collective Drinking	33.8	22.1	31.5	61.3	35.1	31.5	32.9
	Experience of Involuntary One-Shot of Drinks	35.9	36.8	32.4	38.7	30.9	25.3	32.7
	Experiences of Involuntary Drinking ‘Bomb Cocktails’†	26.9	21.1	16.7	25.8	24.5	14.9	22.4
	Experiences of Involuntary Sharing, Exchanging, or Cascading Glassful Drinks	18.8	14.7	13.9	9.7	11.7	9.7	15.0

† a glass of the cocktail containing more than two kinds of alcoholic liquors,

*** *P*<.001 (Mantel-Hanszel x^2^ test was carried out after controlling sex)

One-third of subjects provided responses about their experience related to ‘Passive and Reluctant Participation in Collective Drinking’ and the ‘Experience of One-Shot of Glassful Drinks’. For the experience related to drinking of ‘Bomb Cocktails (a glass of the cocktail containing more than two kinds of alcoholic liquors)’, the frequency was 22%. For the experience involving ‘Coercive or Involuntary Sharing, Exchanging, or Cascading Glassful Drinks’, the frequency was 15.0% among replies from subjects.

Subjects who replied their occupations as workers in ‘Production or Transportation’ industry had the highest portions of the following experiences: 61.3% for ‘Passive and Reluctant Participation in Collective Drinking’ and 38.7% for ‘Experience of One-Shot of Glassful Drinks’. Subjects with occupations of ‘Professionals/Sedentary Workers’ had the following results: 26.9% for drinking of ‘Bomb Cocktails’ and 18.8% for ‘Coercive or Involuntary Sharing, Exchanging, or Cascading Glassful Drinks’. These frequencies were relatively higher than those of subjects with other occupations.

## Discussion

The inquiry delved into the most frequent time of drinking. Based on replies from subjects, the time after 6 o’clock in the evening after completion of daily work was found to be the most frequent time used for drinking. This implies that most drinking behaviors can be counted as one’s leisure activities. One’s daily life is comprised of working and leisure time. The culture of giving reception to one’s guests (with drinking) can also be counted as a bridge that connects both working and leisure in one’s daily life. Subjects with occupations of ‘Directors/Managers’ appeared to have more frequent experiences of ‘Drinking at Receptions’ than subjects in other categories of occupations. This implies another aspect of drinking as one is transformed into different labor beyond its typically recognized conception as one’s behavior to relieve fatigue accumulated by respective labors (1); Thus, the occasion of ‘Drinking at Receptions’ can be regarded as an instance of infiltration of alcoholic drinking into the domain of “labor”. The inquiry delved into the number of drinking sessions showed the following results: the portion of single drinking round was 30%, the portion of the round continued to another additional round was 55.5%, and the portion of three more drinking rounds was 14.5%. This implies that more than half of the initial sessions of drinking are accompanied by subsequent (secondary or tertiary) sessions of drinking. It has to do with the Korean cultural concept of ‘jeong’ which makes people more generous to those who are close to them, even in the professional world. Obviously this is creating all sorts of problems. The traditional culture of the virtues ‘jeong’ is being used as alcohol misuse.

The culture of drinking in our domestic society shows the dominance of types of drinking with purposes of promoting relationships or developing acquaintance with other participants. These were also identified in this study. Frequent occasions of drinking with purposes of developing relationships with others were thus identified, with occasions of ‘Drinking when Dining with Friends or Acquaintances etc.’ and ‘Drinking when Dining with Colleagues’ being the most frequent. In such occasions of drinking, ‘exchanging glassful drinks”, “the ritual of drinking alcoholic drinks in a bowl”, “drinking of bomb cocktails”, or “the practice of one-shot drinking” would be counted as a means of “the culture of collective drinking”.

One-third of subjects were found to have respective experiences of one-shot drinking (complete drinking of glassful drinks at once), with experiences of drinking of bomb cocktail (22.0%) and (coercive or involuntary) sharing, exchanging, or cascading of glassful drinks (15.0%). Coercive or involuntary sharing, exchanging, or cascading of glassful drinks has been pointed out as evil practice of long standing. It requires participants in drinking occasions to drink alcohol with one-shot rather than to drink them sip after sip. Following the one-shot drink, next participants are offered to take subsequent one-shot drink with the empty glass handed over to them. This is a scene frequently found in occasions of drinking held in Korea. According to results obtained from one previous survey, the dominant reason behind the taking or offering of the one-shot drink was the ‘intention of participants to get intoxicated as soon as possible’ (13). In the inquiry delved into reasons behind the taking or offering of one-shot drink of bomb-cocktail, the reason ‘for fun’ was found to be dominant, followed by the reason of ‘intention to get intoxicated as soon as possible’. The Ministry of Food and Drug Safety conducted a national survey on ‘Realities of Drinking’ with 2000 domestic male and female subjects aged over 15 yr living in 17 districts of domestic cities and provinces in 2014. Results obtained thereof revealed 55.8% of respondents had experiences of bomb-cocktail drinking, showing a significant increase from 32.8% of subjects who experienced the same occasions in 2012. However, the portion of subjects experienced drinking of bomb-cocktail was 22.0% in this study. This was somehow lower than those results presented by the Ministry of Food and Drug Safety (14).

Recently, the case of drinking alone has been increasing due to the desire of people to take preferred drinks. This trend was also identified in this study (14). The portion of subjects with experiences of drinking alone was 29.3%. For the occasion of drinking alone, the average amount of alcoholic drinks consumed was found to be 2.7 drinks (one drink=8 g of pure ethanol).

According to results obtained from the ‘Survey of the Hours of Living’ conducted by National Statistical Office in 2014, domestic adults aged over 20 yr enjoyed approximately 4 h and 52 min of daily leisure time (male: 5 h, female: 4 h and 44 min), with watching TVs or being with media occupied approximately 2 h and 28 min, followed by 42 min (male: 37 min, female: 47 min) of the time spend for social occasions with friends, colleagues, and so on. The composition of leisure time of domestic people shows no particularity of hobbies or preferences. Except for the time consumed for the use of mass media, the time allocated to social occasions with friends or others had the highest portion. Besides, most subjects participated in this survey replied that they would usually “participate in occasions of drinking”. This corresponded to the result obtained from this study showing that subjects would participate in occasions of drinking, including drinking at receptions of respective important guests after completing daily work.

The culture of drinking in our society can be considered as one of collective cultures wherein every participant would drink alcoholic drinks together in perfect order. Representative means employed for cultural practices thereof include ‘exchanging of drinks”, “the ritual of drinking alcoholic drinks in a bowl”, “drinking of bomb cocktails”, and “the practice of one-shot drink” requiring every participant to avoid drinking of glassful drinks sip after sip. Coercive offers of one-shot drink to other participants sharing the occasion of drinking would lead participants to have consequential binge drinking (13).

## Conclusion

The culture of drinking could lead them directly to the state of intoxication. One-shot drink of alcohol typically increases blood alcohol concentration rapidly. This frequently leads people to have various harmful consequences. It may cause suffocation due to vomiting or acute alcoholism typically accompanying comatose state or lethality, with highly probable experience of black-out. The black-out may lead patients to physical damages, including reduced capabilities of judgment, impaired impulse control, reduced retentive memory, and secondary ill effects involving violence or robberies; Thus, drinking behavior involving the offering of one-shot drinking needs to be changed. Participants in drinking occasions should be encouraged to be moderate in respective drinking of alcoholic drinks without coercive offers for one-shot drink.

## Ethical considerations

Ethical issues (Including plagiarism, informed consent, misconduct, data fabrication and/or falsification, double publication and/or submission, redundancy, etc.) have been completely observed by the authors.
